# Supplementary material for: Laminarin Reduces Cholesterol Uptake and NPC1L1 Protein Expression in High-Fat Diet (HFD)-Fed Mice
Source: Mar Drugs. 2023 Nov 29;21(12):624. doi: 10.3390/md21120624 (PMC10744832; doi:10.3390/md21120624)
Supplement: Supplementary file 1 [file marinedrugs-21-00624-s001.zip › marinedrugs-2713637-supplementary.pdf]

Supplementary Material

Article

Laminarin Reduces Cholesterol Uptake and NPC1L1 Protein Expression in High-fat Diet (HFD)-fed Mice

Zhuoqian He [1], Zhongyin Zhang [2], Pengfei Xu [1], Verena M. Dirsch [3], Limei Wang [1,4,*] and KeWei Wang [1,4]

[1] Department of Pharmacology, School of Pharmacy, Qingdao University Medical College, Qingdao 266073, Shandong, China

[2] Department of Medicinal Chemistry, School of Pharmacy, Qingdao University Medical College, Qingdao 266073, Shandong, China

[3] Department of Pharmaceutical Sciences, Faculty of Life Sciences, University of Vienna, Josef-Holaubek-Platz 2, 1090 Vienna, Austria

[4] Institute of Innovative Drugs, Qingdao University, Qingdao 266071, Shandong, China

* Correspondence: Limei Wang (limei.wang@qdu.edu.cn)

Supplementary data

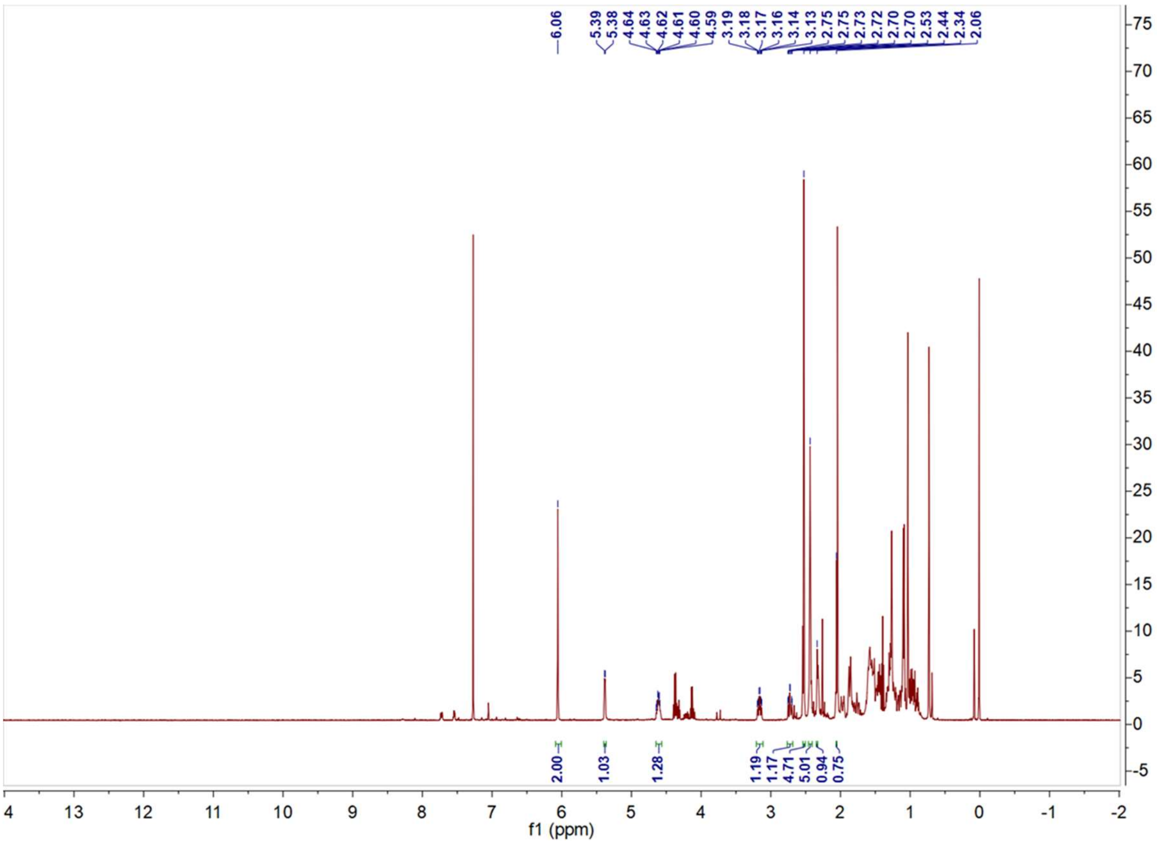

Fig. S1 The NMR data of synthesized fluorescent BODIPY-cholesterol.

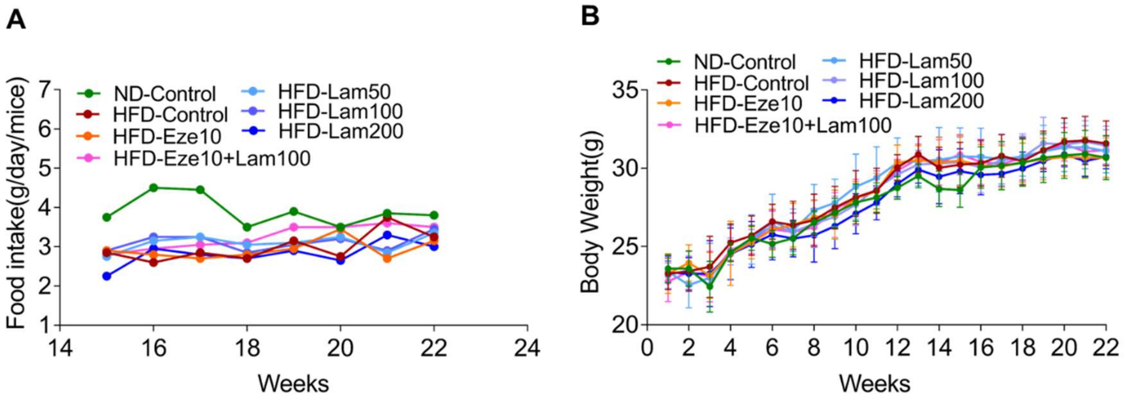

Fig. S2 The food intake and body weight during the experimental procedure. A. The average food intake (g/day/mice) of mice during 15-22 weeks. B. Time-course of body weight in the seven groups of mice. Data were presented as the mean ± S.D. (n=5~12). There was no significance among the different groups (Two-way ANOVA).

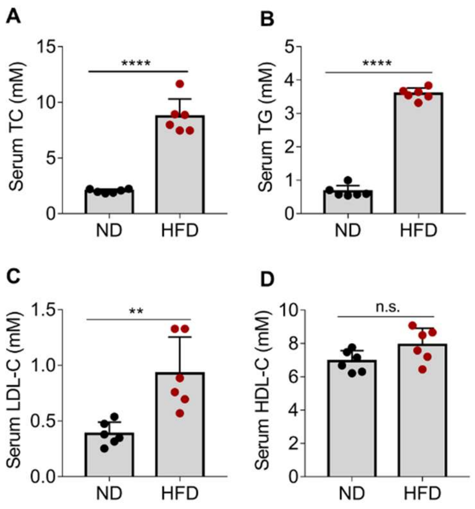

Fig. S3 Comparison of serum lipid profile between HFD mice and ND mice in 17 weeks old male C57BL/6J mice. A. Plasma TC concentration (in mM, n = 6). B. Plasma TG concentration (in mM, n = 6). C. Plasma LDL-Cholesterol concentration (in mM, n = 6). D. Plasma HDL-Cholesterol concentration (in mM, n = 6). The data were presented as the mean ± S.D., $^{**}p < 0.01$, $^{****}p < 0.0001$ (Student's *t* test).

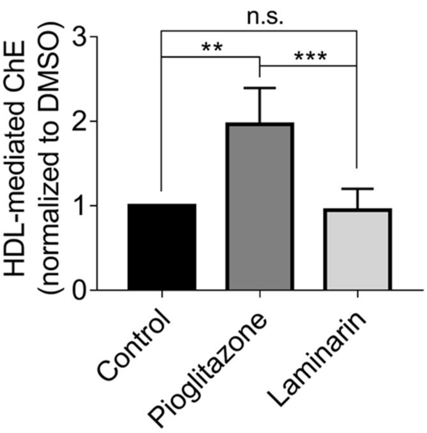

Fig. S4 The effect of lanimarin on cholesterol efflux from J774A.1 cells. J774A.1 macrophages were first labeled with fluorescent BODIPY-cholesterol (0.0625 mM) and loaded with solvent control (DMSO), laminarin (10 μM), and the positive control, pioglitazone (1μM). After 24 h incubation, cells were washed twice with PBS and incubated again with the same compounds in the presence or absence of 1% human plasma dissolved in serum-free medium for 6 h. Extracellular as well as intracellular fluorescence intensity were quantified by flexstation 3. The data were presented as the mean ± S.D., **$p < 0.01$, ***$p < 0.001$, *n.s.* no significance (One-way ANOVA).
